# Temporal trends in TB notification rates during ART scale-up in Cape Town: an ecological analysis

**DOI:** 10.7448/IAS.18.1.20240

**Published:** 2015-09-25

**Authors:** Sabine Hermans, Andrew Boulle, Judy Caldwell, David Pienaar, Robin Wood

**Affiliations:** 1 Desmond Tutu HIV Centre, Institute of Infectious Disease and Molecular Medicine, University of Cape Town, Cape Town, South Africa; 2 Department of Global Health, Academic Medical Center, University of Amsterdam, Amsterdam Institute for Global Health and Development, Amsterdam, The Netherlands; 3 Department of Internal Medicine, School of Medicine, Makerere University College of Health Sciences, Kampala, Uganda; 4 School of Public Health and Family Medicine, University of Cape Town, Cape Town, South Africa; 5 Western Cape Government, Department of Health, Cape Town, South Africa; 6 City Health, Department of Health, City of Cape Town, South Africa; 7 Department of Medicine, University of Cape Town, Cape Town, South Africa; 8 Department of Clinical Research, Faculty of Infectious & Tropical Diseases, London School of Hygiene & Tropical Medicine, London, UK

**Keywords:** HIV, tuberculosis, antiretroviral therapy, population impact, South Africa

## Abstract

**Introduction:**

Although antiretroviral therapy (ART) reduces individual tuberculosis (TB) risk by two-thirds, the population-level impact remains uncertain. Cape Town reports high TB notification rates associated with endemic HIV. We examined population trends in TB notification rates during a 10-year period of expanding ART.

**Methods:**

Annual Cape Town TB notifications were used as numerators and mid-year Cape Town populations as denominators. HIV-stratified population was calculated using overall HIV prevalence estimates from the Actuarial Society of South Africa AIDS and Demographic model. ART provision numbers from Western Cape government reports were used to calculate overall ART coverage. We calculated rates per 100,000 population over time, overall and stratified by HIV status. Rates per 100,000 total population were also calculated by ART use at treatment initiation. Absolute numbers of notifications were compared by age and sub-district. Changes over time were described related to ART provision in the city as a whole (ART coverage) and by sub-district (numbers on ART).

**Results:**

From 2003 to 2013, Cape Town's population grew from 3.1 to 3.7 million inhabitants, and estimated HIV prevalence increased from 3.6 to 5.2%. ART coverage increased from 0 to 63% in 2013. TB notification rates declined by 16% (95% confidence interval (CI), 14–17%) from a 2008 peak (851/100,000) to a 2013 nadir (713/100,000). Decreases were higher among the HIV-positive (21% (95% CI, 19–23%)) than the HIV-negative (9% (95% CI, 7–11%)) population. The number of HIV-positive TB notifications decreased mainly among 0- to 4- and 20- to 34-year-olds. Total population rates on ART at TB treatment initiation increased over time but levelled off in 2013. Overall median CD4 counts increased from 146 cells/µl (interquartile range (IQR), 66, 264) to 178 cells/µl (IQR 75, 330; *p*<0.001). Sub-district antenatal HIV seroprevalence differed (10–33%) as did numbers on ART (9–29 thousand). Across sub-districts, infant HIV-positive TB decreased consistently whereas adult decreases varied.

**Conclusions:**

HIV-positive TB notification rates declined during a period of rapid scale-up of ART. Nevertheless, both HIV-positive and HIV-negative TB notification rates remained very high. Decreases among HIV positives were likely blunted by TB remaining a major entry to the ART programme and occurring after delayed ART initiation.

## Introduction

Antiretroviral therapy (ART) reduces the individual tuberculosis (TB) risk by two-thirds [1]. This reduction is time- and CD4+ T-cell (CD4) count-dependent, however [2]. The TB risk is highest at low CD4 counts, often just before ART initiation and in the first months on ART due to unmasking of subclinical TB disease, and reduces rapidly within the first two years of ART. Despite good immune recovery on ART, the long-term TB risk remains several fold higher compared to those who are HIV uninfected [3,4]. At a population level, mathematical models have predicted substantial reductions of HIV-associated TB rates with increasing ART coverage [5–8]. The amount and sustainability of the reductions vary substantially between models, highlighting the uncertainty around these estimates.

South Africa has the highest TB notification rate in the world after Swaziland and Lesotho, and the Cape Town district reports the second highest number of TB notifications in the country [9,10]. Cape Town also has a generalized HIV epidemic with antenatal HIV seroprevalence estimated at 19.7% [11]. ART became available in the public sector in 2001. The city started roll-out of ART for all HIV-infected individuals with a CD4 count below 200 cells/µl after the launch of the national programme in 2004 [12]. This eligibility threshold was raised to 350 cells/µl in 2012 and to 500 cells/μl in 2015 [13,14]. Since the start of the programme, more than 130,000 people have been initiated on ART [15]. We examined population trends in TB notification rates by HIV disease status during a 10-year period of increasing ART coverage.

## Methods

### Setting and data sources

ART is provided by 48 clinics and hospitals across eight health sub-districts of the Cape Town metropolitan area. TB care is provided by 101 government primary care clinics, of which 40 also provide ART. In January 2013, the primary test in the TB diagnostic algorithm changed from sputum smear microscopy to Xpert MTB/RIF [16].

The annual numbers of TB notifications in the Cape Town metropolitan area between 2003 and 2013 were abstracted from the Electronic TB Register (ETR), overall and per sub-district. ETR.net is an electronic register used to capture TB patient records across South Africa and contains data on age, sex, type of TB, history of TB treatment, results of TB diagnostic tests, HIV test results, ART usage at treatment initiation, CD4 count at treatment initiation and treatment outcome of registered TB notifications (updated until the end of June 2014). To avoid double counting, we excluded notifications that were transferred in from a different clinic or district during TB treatment.

We used the annual mid-year populations of the Cape Town metropolitan area estimated by Statistics South Africa [17]. The annual HIV prevalence in the city of Cape Town was estimated using the Western Cape version of the Actuarial Society of South Africa (ASSA) AIDS and Demographic model 2008 [18]. We calculated the estimated HIV-infected population by applying the annual HIV prevalence to the mid-year population estimate.

The sub-district antenatal HIV seroprevalences were sourced from the annual Western Cape Antenatal Survey reports [19]. Data on the annual numbers of people currently on ART per sub-district were obtained from the annual Western Cape Government District Health Information System [15].

### Statistical methods

ART coverage was calculated as the proportion of the estimated HIV-infected population who were on ART, in accordance with the UNAIDS guidelines to construct core AIDS indicators [20]. These were recently changed from the proportion of estimated ART-eligible HIV-infected population who were on ART to the proportion of all HIV-infected patients to facilitate comparisons across countries and settings with varying ART eligibility criteria.

We calculated annual TB notification rates per 100,000 population (with 95% confidence intervals (CI) using the delta method) [21], by dividing the annual number of TB notifications by the estimated mid-year population. These rates were stratified by HIV status from 2009 onwards, when ≥90% of TB notifications had a recorded HIV status. We extrapolated the HIV prevalence among TB notifications from those tested to those who were not. This allowed us to estimate the total numbers of HIV-positive and HIV-negative TB notifications per year, which were then used to calculate estimated annual HIV-stratified TB notification rates per 100,000 population for the entire period under study. We applied the 2004 HIV prevalence to 2003, as only three TB notifications had a recorded HIV status in that year.

Stratified rates were calculated using stratified numerators and denominators, except for stratification by use of ART at start of TB treatment for which we used the total population as the denominator. This was done for fear of unreliable estimates because of uncertainty of both the numerator and the denominator. For comparative plotting purposes, we also calculated rates per total population by HIV status (actual and estimated).

We calculated the per cent change in TB notification rate per 100,000 population per year, overall, and stratified by HIV status (using the actual stratified rates 2009 to 2013). Median annual CD4 counts over time were compared using Cuzick's non-parametric test for trend, and median CD4 counts on ART and not on ART at start of TB treatment initiation were compared using the Wilcoxon rank sum test.

As reliable population estimates for HIV prevalence were unavailable by five-year age groups and at a sub-district level, rates and ART coverage were not calculated stratified by age group or by sub-district. Only absolute numbers of TB cases stratified by HIV status and of ART usage were compared. We calculated and compared the average annual reduction (absolute and relative) in the number of HIV-positive and HIV-negative TB notifications over time by five-year age groups.

The analyses were performed using STATA 13.0 IC (College Station, Texas) and Microsoft Excel 2013 (Redmond, Virginia). All statistical tests were two-sided at an *α* value of 0.05.

### Ethics approval

The Human Research Ethics Committee of the University of Cape Town considered this study exempt from ethical review as our study only used anonymized data in aggregate.

## Results

In total, 295,615 patients were initiated on TB treatment between 2003 and 2013. Of these, 55% were male with a median age of 31 years (interquartile range (IQR), 23, 41) and 26% had been treated for TB before. The proportion of TB notifications that were microbiologically confirmed (by sputum smear microscopy, sputum culture or Xpert MTB/RIF) was 59%. Baseline characteristics by year of treatment initiation are summarized in Supplementary Table 1. Overall treatment success (defined as completed treatment or cured) was 78%, increasing from 74% in 2003 to 83% in 2012. Treatment outcomes for 2013 were not yet complete. Recorded HIV status of TB notifications increased from 0% in 2003 to 93% in 2009, further increasing to 98% in 2012 and 2013 (Table 1 and Supplementary Figure 1). In the years with near-complete reporting of HIV status (≥2009), 47% were HIV infected. The proportion of HIV positive among those with known HIV status remained relatively stable over time (44–50%), suggesting the HIV prevalence among those untested in earlier years was similar.

**Table 1:** TB notification rates per 100,000 population and annual per cent change in rates over time, overall and stratified by HIV status

	Total			HIV negative		HIV positive	
			
Year	TB notification rate (95% CI)^a^	% rate change	Reported HIV status (%)	TB notification rate (95% CI)^a^	% rate change	TB notification rate (95% CI)^a^	% rate change
2003	747 (738–757)		0.0				
2004	769 (759–779)	2.9	3.2				
2005	824 (814–834)	7.2	48.1				
2006	809 (799–819)	−1.8	65.9				
2007	800 (790–809)	−1.2	66.0				
2008	851 (841–861)	6.4	78.7				
2009	843 (833-852)	−1.0	92.6	422 (415–429)		7825 (7692–7960)	
2010	845 (835–855)	0.3	96.4	454 (446–461)	7.4	7767 (7637–7900)	−0.7
2011	799 (790–809)	−5.4	97.4	435 (428–443)	−4.0	7306 (7182–7432)	−5.9
2012	756 (748–766)	−5.4	97.7	422 (415–429)	−3.1	6716 (6598–6836)	−8.1
2013	713 (704–721)	−5.8	98.4	412 (406–419)	−2.2	6119 (6007–6232)	−8.9

Rate changes stratified by HIV status were only calculated for the years that more than 90% of notifications had a recorded HIV status (2009 to 2013). CI, confidence interval.

a Rate per 100,000 population.

**Figure 1:**
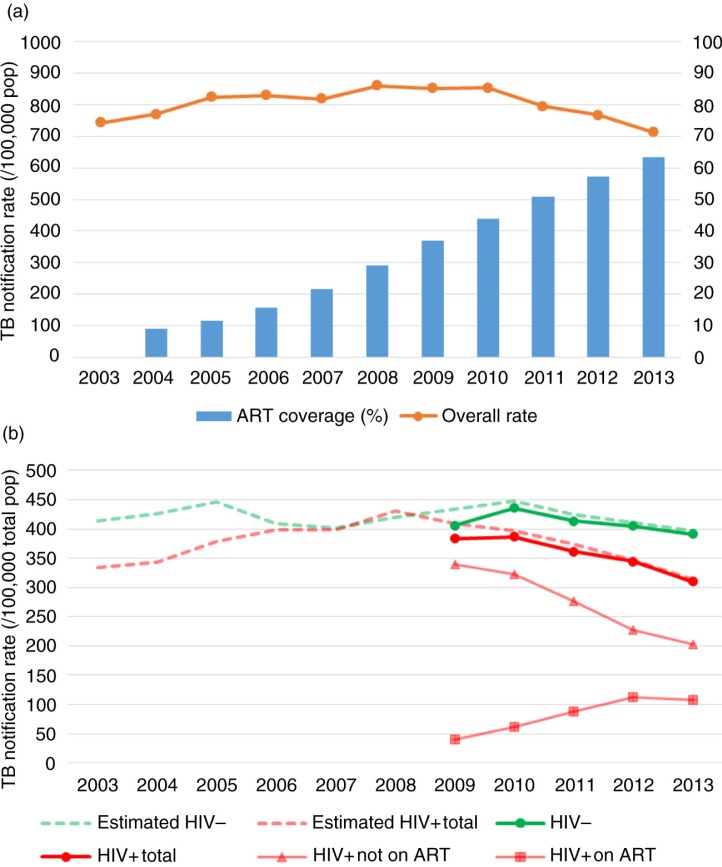
(a) Overall TB notification rates and ART coverage in the estimated HIV-positive population over time. (b) TB notification rates per total population over time, overall, and stratified by HIV status and by ART use at start of TB treatment. The number of HIV-positive and HIV-negative TB notifications pre 2009 were estimated using the proportion of HIV positivity among the TB notifications with a known HIV status. Note: HIV−, HIV negative; HIV+, HIV positive.

The estimated mid-year population of Cape Town increased from 3.1 million in 2003 to 3.7 million in 2013. During the same period, the estimated HIV prevalence increased from 3.6 to 5.2%. In 2003, 11,058 (9%) of 121,597 estimated HIV-infected persons were on ART. By the end of 2013, 118,262 (63%) out of an estimated 186,658 were on ART (Figure 1a). Numbers of people on ART varied by sub-district, ranging from 8765 in the Northern sub-district to 28,738 in the Khayelitsha sub-district in 2013.

TB notification rates slowly increased to reach a plateau at around 850 per 100,000 population between 2008 and 2010, then declined in the three subsequent years (Table 1 and Figure 1a). Total annual numbers of TB notifications and mid-year population estimates, overall and stratified by HIV status, are shown in Supplementary Table 2. Overall, TB notification rates decreased by 15.6% (95% CI, 14.2–17.1%) between 2010 and 2013. This reduction was seen in both HIV positives (21.2% (95% CI, 19.3–23.1%)) and HIV negatives (9.1% (95% CI, 7.0–11.1%)). Annual rate changes varied up to 2010 but showed a consistent decrease from 2011 onwards (Table 1). Annual decreases were less among HIV negatives than HIV positives. The total population rates stratified by HIV status and by use of ART at the start of treatment are shown in Figure 1b. The proportion of TB notification rates on ART increased over time, but this increase levelled off in the most recent year.

Of the TB notifications known to be HIV positive, the median CD4 count at TB diagnosis (available in 95% of HIV-positive notifications) increased from 153 cells/µl (IQR 70, 283) in 2009 to 178 cells/µl (IQR 75, 330) in 2013 (Supplementary Table 1, *p*<0.001). The proportion of HIV-infected TB notifications reported as presenting while not yet on ART decreased from 89% in 2009 to 66% in 2013. Both the median CD4 count of those on ART at TB treatment initiation and those not on ART increased over time (Supplementary Table 1, *p*<0.001). However, the median CD4 count on ART was higher than the median CD4 count not on ART (178 cells/µl (IQR 81, 309) compared to 166 cells/µl (IQR 73, 308), *p*<0.001).

**Figure 2:**
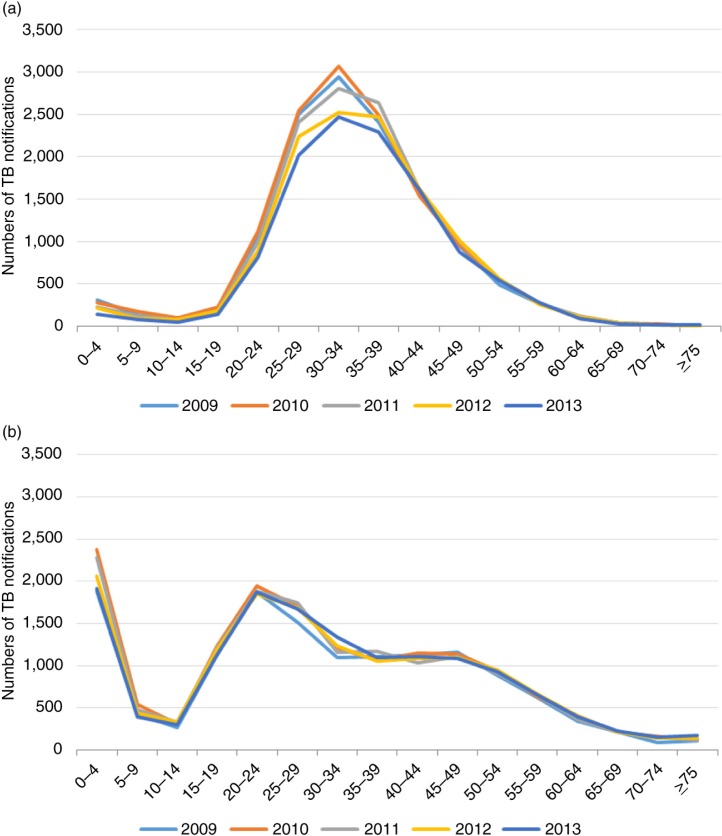
(a) Numbers of HIV-positive notifications per 5-year age group over time. (b) Numbers of HIV-negative notifications per 5-year age group over time.

Numbers of HIV-positive TB notifications and average annual changes stratified by five-year age groups from 2009 to 2013 are shown in Figure 2a and Supplementary Table 3. Reductions were mainly seen among 0- to 4-year-olds (average decrease of 43 notifications per year) and 20- to 34-year-olds (average decrease of 66, 120 and 119 notifications per year, for the 20–24, 25–29 and 30–34 age groups, respectively) between 2009 and 2013. Among HIV-negative TB notifications during the same period, no age-specific reductions were seen, but an increase among 25- to 34-year-olds was seen (Figure 2b and Supplementary Table 3).

**Figure 3:**
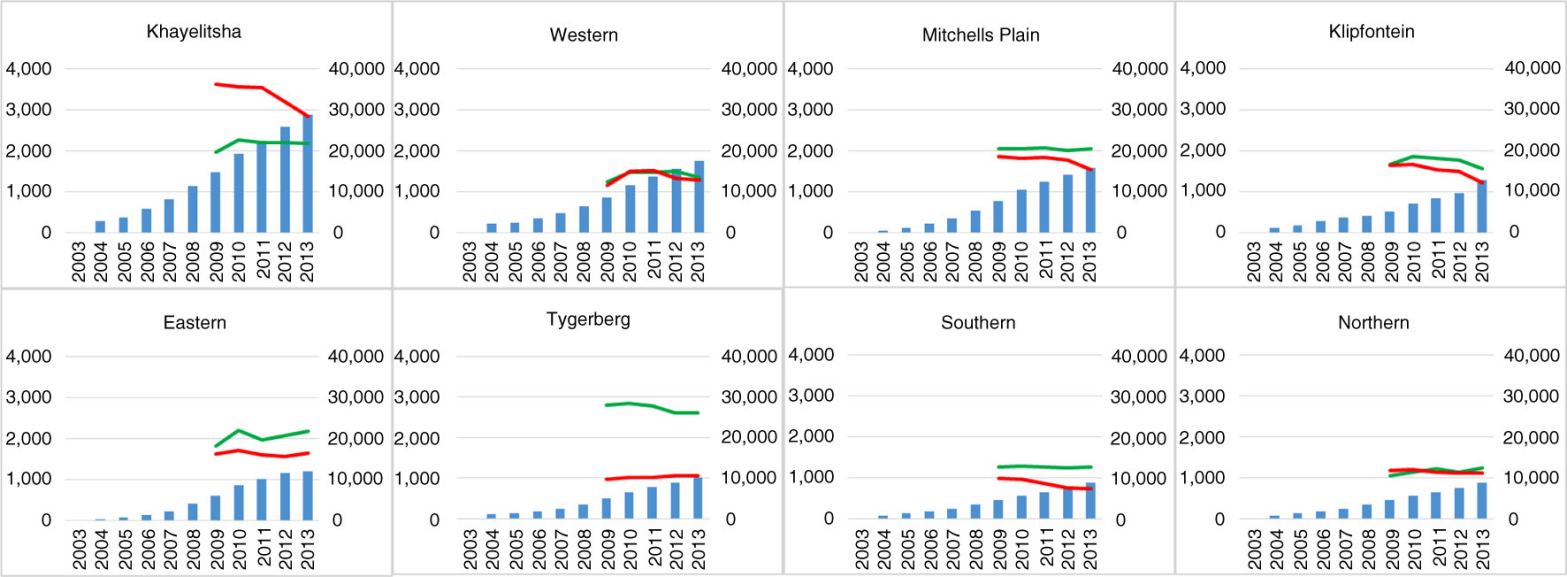
Absolute number of TB notifications per health sub-district over time. Left vertical axis: HIV-negative (green) and HIV-positive (red) TB notifications. The blue bars represent absolute numbers of people on ART (right vertical axis). The health sub-districts are ordered by the total number of people on ART in 2013.

The eight Cape Town health sub-districts encompassed each around 450,000 people, ranging between 330,000 (Northern sub-district) and 560,000 (Tygerberg sub-district; Supplementary Table 4). They varied substantially in antenatal HIV seroprevalence, with the five-year average ranging between 10% (Southern sub-district) and 33% (Khayelitsha sub-district; Supplementary Table 4). The changes in the number of HIV-negative and HIV-positive TB notifications from 2009 to 2013 varied across the sub-districts (Figure 3). Khayelitsha, the sub-district with the highest uptake of ART, showed a sharp decline among HIV positives but not among HIV negatives. Across all sub-districts, annual reductions in HIV-associated TB were seen consistently among infants, whereas the decrease among adults was more variable (Supplementary Figures 2a and b).

## Discussion

HIV care in the city of Cape Town has substantially advanced over the last 10 years, with known HIV status of TB notifications nearing 100% and scale-up of ART coverage increasing from 0 to 63% in nine years. Nevertheless, TB notification rates continue to be very high in this population. After an initial increase and stabilization, TB notification rates declined approximately 16% over the last three years in both the HIV-positive and the HIV-negative population. Decreases in HIV-associated TB notification rates were approximately twice compared to non-HIV-associated TB rates during that period.

Although our ecological study design does not allow for the attribution of these reductions to the increasing ART coverage, the temporality of the changes as well as the concentrated decreases in certain age groups are suggestive of an effect of ART. HIV burden is highest among 25- to 39-year-olds and ART is most often initiated among those in their early thirties [4,22,23]. Furthermore, the reductions among the under-fives could be a manifestation of the success of the prevention of mother to child transmission programme in South Africa [24].

At a sub-district level, with substantial variation in antenatal seroprevalence and uptake of ART, a trend towards greater reductions in the number of HIV-positive TB notifications in sub-districts with higher numbers of people on ART was still discernible. Stratified by age, decreases in HIV-positive TB among infants were consistent whereas those among adults were more variable. Changes in the numbers of HIV-negative TB notifications seemed temporally unrelated, however. These varying patterns could be a reflection of the differential impact of ART in settings with different levels of HIV prevalence. Limitations of this sub-district analysis include the lack of reliable population denominators stratified by HIV status, making calculation of notification rates and ART coverage impossible. Also, access to health services crosses sub-district boundaries, which could lead to the possible attribution of notifications to a different sub-district than where they reside.

In addition to the effect of ART, there could be alternative explanations for the declining TB notification rates. There could have been changes in the TB control programme leading to improved TB control. No new city-wide interventions have taken place, and treatment success rates have remained stably high throughout the period of observation. Ascertainment could have changed, although case finding practices in the city have not changed. Heightened awareness might have led to increased ascertainment among the HIV-positive population, especially those on ART, which would lead temporarily to increased TB notifications among this group. The population denominators underlying the rates we calculated could have been an overestimate of the real population, for which we relied on HIV prevalence derived from a mathematical model developed in 2008 [18]. Last, the force of TB infection could have decreased due to other changes, for example socio-economic improvements. However, we would have expected this to have led to greater reductions in HIV-negative TB, in particular among young children.

Our data illustrate the difference between individual-level and population-level impact. The estimated 60% decrease in individual risk does not necessarily translate into a similar population decrease. Attempts to predict the population-level impact in South Africa have been made by several mathematical models [5–8]. These models have used differing definitions and assumptions of ART coverage levels, CD4 count thresholds defining ART eligibility, and access and retention to care. These varying assumptions make comparisons between the model predictions and our real-life data difficult. With a threshold of <350 cells/µl, used in South Africa up to the end of 2014, the predicted decreases range between 0 and 50% by 2030 [5–8]. A model comparing optimistic and pessimistic scenarios showed very disparate trajectories [7]. The latter model included projections of an increase in rates after an initial fast decrease because of increased life expectancy with increasing use of ART leading to a growing population at risk of recurrent TB [25]. This phenomenon was not yet noticeable during the period under study.

Only few observational data have been reported on the population impact of ART. In 2011, a 20% decrease in overall TB notification rates was reported from a Cape Town township with increasing ART coverage to 20% of the HIV-infected population [26]. A sub-district in Malawi saw new TB rates reduce by 33% and recurrent TB rates by 25% after ART coverage had been scaled up to more than 50% of the eligible population [27]. These data have recently been augmented by data from two sub-Saharan African countries, Kenya and Malawi, which describe substantial decreases in their TB notification rates, among both HIV infected and uninfected [28,29]. Their decreases started at ART coverage levels of around 15 to 20%, whereas declines in Cape Town only started at coverage levels of 30 to 40%. Differences might indicate a threshold effect of ART coverage to achieve impact on TB incidence, possibly dependent on the local burden of HIV and TB disease and the stage of the epidemic. There have also been suggestions that the rate of ART scale-up in the community could determine the impact on TB notification rates [26,30]. The estimated HIV-stratified rates indicated that the decrease in HIV-positive rates might have started earlier than we could conclude on the basis of our original data. Also, our estimates of ART coverage might be inflated by people in need of ART migrating from rural areas to the city because of a higher perceived level of HIV care [31], leading to a higher HIV prevalence than predicted by the ASSA model.

The levelling off of population TB notification rates on ART and the increasing median CD4 count of TB patients on ART suggest that the population of TB patients presenting after ART initiation is changing. This could be explained by earlier ART initiation over time as well as increasing numbers on ART for a longer duration [32]. Nevertheless, the risk of TB is highly associated with low CD4 counts even in those on ART [2]. An increase in community CD4 count due to increased ART coverage might not necessarily translate into HIV-associated TB occurring at higher CD4 counts, but rather that less HIV-associated TB would occur but still at low CD4 counts. The still low median CD4 count of TB patients on ART may be indicative that these notifications are occurring in newly started patients accessing ART late, leading to unmasking of prevalent TB at the start of ART [33,34]. Further research is needed to evaluate this in more detail.

The proportion of HIV-positive TB notifications initiating treatment not on ART is still high. This corresponds with data from an observational ART cohort in Cape Town where 35% of patients were referred to the ART programme after a diagnosis of TB [35]. Entry into the programme at the time of TB diagnosis is a barrier to the impact of ART. Until a substantially larger proportion of HIV-infected patients are initiated on ART before presenting to the TB control programme, it is unlikely that increased ART coverage will reach its maximal impact.

Limitations of our analysis include the possible misclassification of ART usage at initiation of TB treatment among HIV-positive notifications, which would underestimate the proportion of TB notifications presenting after ART initiation and bias our median CD4 count estimates. We lacked data on ART start dates, which would have allowed us to explore the contribution of unmasking prevalent TB. We applied the HIV prevalence in the Western Cape ASSA model to the population of Cape Town, which might be an underestimate.

## Conclusions

HIV-positive TB notification rates declined during a period of rapid scale-up of ART. Nevertheless, both HIV-positive and HIV-negative TB notification rates remained very high throughout the study period. Decreases among the HIV positives were likely blunted by TB remaining a major entry to the ART programme and occurring in patients accessing ART late in the course of their HIV disease. Increased access to ART before the onset of TB will be essential to maximize the impact of ART and to achieve TB control in Cape Town.

## Supplementary Material

Temporal trends in TB notification rates during ART scale-up in Cape Town: an ecological analysis
