# Supplementary material for: Temporal trends in TB notification rates during ART scale-up in Cape Town: an ecological analysis
Source: J Int AIDS Soc. 2015 Sep 25;18(1):20240. doi: 10.7448/IAS.18.1.20240 (PMC4584214; doi:10.7448/IAS.18.1.20240)

SUPPLEMENTARY MATERIAL

Table S1. Baseline characteristics over time.

Characteristic (n[%])	2003	2004	2005	2006	2007	2008	2009	2010	2011	2012	2013
**Total**	**22,821**	**23,993**	**26,271**	**26,340**	**26,508**	**28,744**	**29,011**	**29,641**	**28,557**	**27,463**	**26,266**
**Female gender**	9660 (42.3)	10414 (43.4)	11317 (43.1)	11832 (44.9)	11972 (45.2)	13251 (46.1)	13149 (45.3)	13910 (46.9)	13096 (45.9)	12363 (45)	11810 (45)
**Age (median [IQR])**	31 (22,41)	31 (22,40)	31 (22,41)	31 (22,41)	31 (22,41)	32 (23,41)	32 (23,41)	31 (23, 41)	32 (23, 41)	32 (23, 42)	33 (24, 42)
**Retreatment**	5909 (25.9)	6114 (25.5)	6901 (26.3)	6931 (26.3)	7204 (27.2)	7646 (26.6)	7534 (26)	7534 (25.4)	7401 (25.9)	7350 (26.8)	6271 (23.9)
**Microbiological confirmation**	14455 (63.3)	15179 (63.3)	16393 (62.4)	15735 (59.7)	15520 (58.5)	15895 (55.3)	15790 (54.4)	16118 (54.4)	15899 (55.7)	16198 (59)	16344 (62.2)
**HIV negative**	2 (0)	432 (1.8)	6883 (26.2)	8937 (33.9)	8879 (33.5)	11300 (39.3)	13832 (47.7)	15125 (51)	14781 (51.8)	14539 (52.9)	14428 (54.9)
**HIV positive**	1 (0)	348 (1.5)	5850 (22.3)	8693 (33)	8794 (33.2)	11615 (40.4)	13045 (45)	13441 (45.3)	13044 (45.7)	12282 (44.7)	11421 (43.5)
**HIV unknown**	22818 (100)	23213 (96.7)	13538 (51.5)	8710 (33.1)	8835 (33.3)	5829 (20.3)	2134 (7.4)	1075 (3.6)	732 (2.6)	642 (2.3)	417 (1.6)
**CD4 count reported among HIV+**	1 (100)	0 (0)	0 (0)	3 (0)	357 (4.1)	7827 (67.4)	11929 (91.4)	12859 (95.7)	12634 (96.9)	11856 (96.5)	10990 (96.2)
**CD4 count at TB diagnosis* (median [IQR])**	107 (107, 107)	-	-	56 (26, 572)	141 (73, 275)	146 (66, 264)	153 (70, 283)	162 (74, 293)	176 (79, 313)	181 (76, 326)	178 (75,330)
**On ART at TB diagnosis among HIV+**	0 (0)	2 (0.6)	44 (0.8)	67 (0.8)	131 (1.5)	1420 (12.2)	1383 (10.6)	2139 (15.9)	3139 (24.1)	4055 (33.0)	3944 (34.5)
**CD4 count on ART (median [IQR])**	-	-	-	56 (56, 56)	83 (41, 145)	120 (51, 201)	149 (70, 247)	155 (73, 257)	174 (81, 290)	185 (81, 325)	202 (91, 351)
**CD4 count not on ART (median [IQR])**	107 (107, 107)	-	-	299 (26, 572)	157 (81, 291)	151 (70, 273)	154 (70, 286)	164 (74, 300)	176 (78, 320)	178 (74, 326)	164 (67, 318)

Note. IQR, interquartile range; n, number; TB, tuberculosis.

[#]by sputum smear microscopy, sputum culture or Xpert MTB/RIF (the latter from October 2012 onwards).

Table S2. Annual numbers of TB notifications and mid-year population, overall and stratified by HIV status.

	Total		HIV negative		HIV positive	
**Year**	**TB notifications**	**Population**	**TB notifications**	**Population**	**TB notifications**	**Population**
2003	22,821	3,054,122	2	2,944,990	1	109,132
2004	23,993	3,121,057	432	2,999,460	348	121,597
2005	26,271	3,187,720	6,883	3,054,939	5,850	132,781
2006	26,340	3,256,301	8,937	3,113,209	8,693	143,092
2007	26,508	3,315,371	8,879	3,163,724	8,794	151,647
2008	28,744	3,378,192	11,300	3,218,944	11,615	159,248
2009	29,011	3,443,010	13,832	3,276,293	13,045	166,717
2010	29,641	3,507,635	15,125	3,334,586	13,441	173,049
2011	28,557	3,572,919	14,781	3,394,379	13,044	178,540
2012	27,463	3,630,272	14,539	3,447,395	12,282	182,877
2013	26,266	3,686,266	14,428	3,499,613	11,421	186,658

Table S3. Numbers of TB notifications by 5 year age-group and average absolute and relative annual change stratified by HIV status, 2009-2013.

Age (years)	2009	2010	2011	2012	2013	Change (absolute)	Change (%)	2009	2010	2011	2012	2013	Change (absolute)	Change (%)
0-4	310	273	225	216	137	-43	-17.5	1,878	2,378	2,281	2,059	1,911	8.3	1.4
5-9	132	169	122	83	72	-15	-11.3	428	535	471	436	395	-8.3	-0.9
10-14	76	103	72	74	50	-7	-6.1	261	311	331	331	299	9.5	4.0
15-19	191	222	178	183	137	-14	-6.5	1,130	1,231	1,206	1,194	1,131	0.3	0.2
20-24	1,075	1,113	992	890	810	-66	-6.7	1,877	1,944	1,887	1,852	1,869	-2.0	-0.1
25-29	2,502	2,540	2,403	2,240	2,021	-120	-5.1	1,511	1,708	1,747	1,680	1,675	41.0	2.8
30-34	2,945	3,068	2,810	2,519	2,469	-119	-4.1	1,095	1,208	1,160	1,231	1,341	61.5	5.4
35-39	2,406	2,495	2,635	2,471	2,297	-27	-1.0	1,107	1,066	1,167	1,057	1,099	-2.0	0.1
40-44	1,557	1,534	1,607	1,621	1,605	12	0.8	1,125	1,149	1,033	1,086	1,111	-3.5	-0.1
45-49	948	956	1,005	1,016	875	-18	-1.7	1,159	1,135	1,101	1,100	1,083	-19.0	-1.7
50-54	484	546	548	555	534	13	2.7	889	939	903	944	923	8.5	1.0
55-59	264	273	268	260	275	3	1.1	626	632	654	662	654	7.0	1.1
60-64	104	85	116	107	84	-5	-2.8	342	398	360	405	396	13.5	4.3
65-69	34	33	36	31	28	-2	-4.4	211	215	211	217	224	3.3	1.5
70-74	11	25	17	9	16	1	31.5	83	156	146	140	148	16.3	20.8
≥75	6	6	10	7	11	1	23.5	110	120	123	145	169	14.8	11.5
Total	13,045	13,441	13,044	12,282	11,421	-406	-3.2	13,832	15,125	14,781	14,539	14,428	149.0	1.2

Table S4. Average population and antenatal HIV sero-prevalence per health sub-district, in order of numbers of people on ART in 2013.

Average 2009-2013	Khayelitsha	Western	Mitchells Plain	Klipfontein	Eastern	Tygerberg	Southern	Northern
Population (n)	398568	402591	487666	423761	416807	563044	533387	332215
Antenatal sero-prevalence (%)	0.34	0.20	0.17	0.23	0.18	0.11	0.10	0.22

Figure S1. Known HIV status among TB cases and HIV prevalence among those with known HIV status over time.

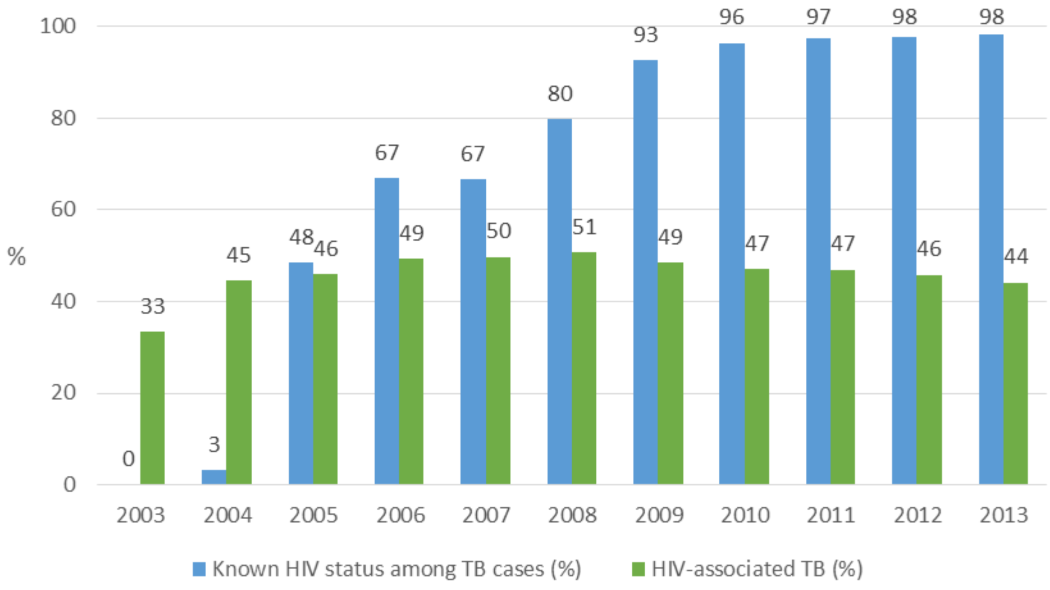

Figure S2a. Absolute numbers of HIV positive TB cases per health sub-district by year and by 5-year age group. The health sub-districts are in decreasing order of the number of people on ART in 2013.

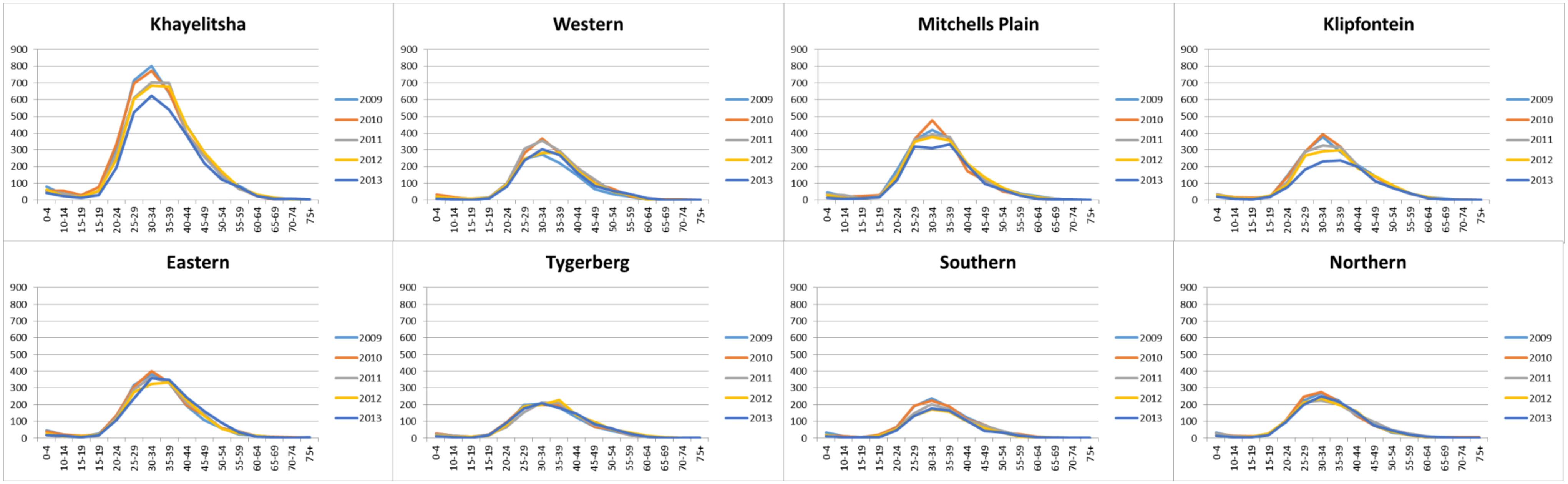

Figure S2b. Absolute numbers of HIV negative TB cases per health sub-district by year and by 5 year age group. The health sub-districts are in decreasing order of the number of people on ART in 2013.

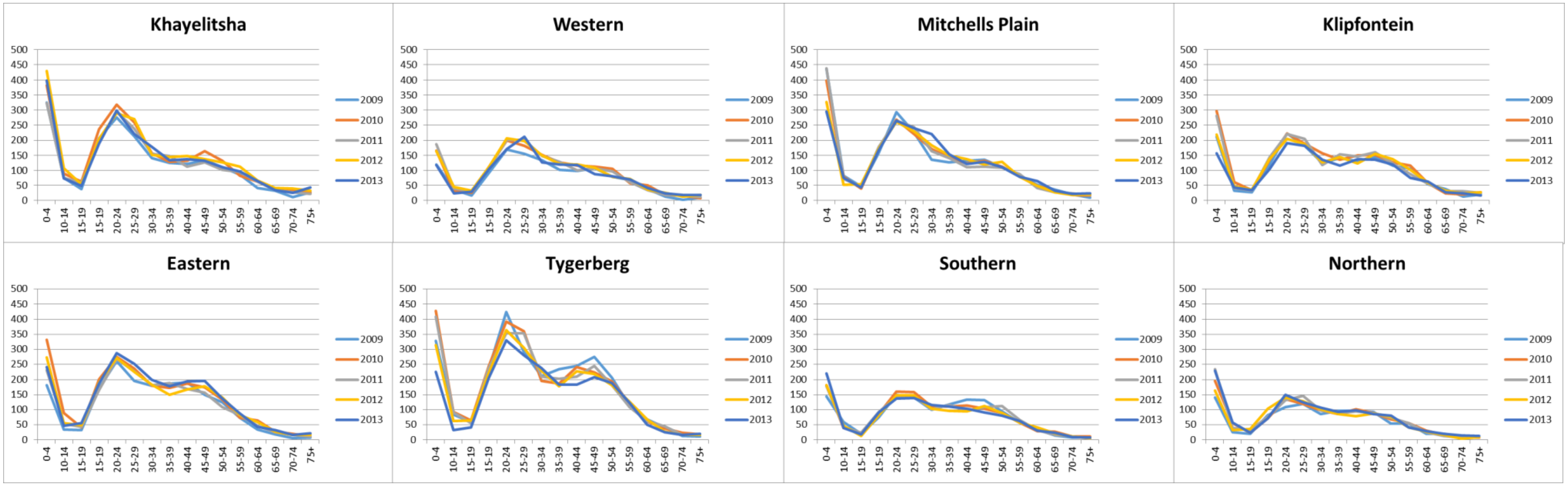

Supplement: Temporal trends in TB notification rates during ART scale-up in Cape Town: an ecological analysis [file JIAS-18-20240-s001.pdf]
